# Kinetic of Adhesion of *S. epidermidis* with Different EPS Production on Ti6Al4V Surfaces

**DOI:** 10.1155/2019/1437806

**Published:** 2019-11-26

**Authors:** Miguel Ángel Pacha-Olivenza, Abraham Rodríguez-Cano, M. Luisa González-Martín, Amparo M. Gallardo-Moreno

**Affiliations:** ^1^Department of Biomedical Sciences, Faculty of Medicine, University of Extremadura, Avda de Elvas s/n, Badajoz 06006, Spain; ^2^Networking Research Center on Bioengineering, Biomaterials and Nanomedicine (CIBER-BBN), Badajoz, Spain; ^3^University Institute of Biosanitary Research of Extremadura (INUBE), Badajoz 06006, Spain; ^4^Department of Applied Physics, Faculty of Science, University of Extremadura, Avda de Elvas s/n, Badajoz 06006, Spain

## Abstract

Controlling initial bacterial adhesion is essential to prevent biofilm formation and implant-related infection. The search for surface coatings that prevent initial adhesion is a powerful strategy to obtain implants that are more resistant to infection. Tracking the progression of adhesion on surfaces from the beginning of the interaction between bacteria and the surface provides a deeper understanding of the initial adhesion behavior. To this purpose, we have studied the progression over time of bacterial adhesion from a laminar flow of a bacterial suspension, using a modified Robbins device (MRD). Comparing with other laminar flow devices, such as the parallel plate flow chamber, MRD allows the use of diverse substrata under the same controlled flow conditions simultaneously. Two different surfaces of Ti6Al4V and two strains *of Staphylococcus epidermidis* with different exopolymer production were tested. In addition, the modified Robbins device was examined for its convenience and suitability for the purpose of this study. Results were analyzed according to a pseudofirst order kinetic. The values of the parameters obtained from this model make it possible to discriminate the adhesive behavior of surfaces and bacteria. One of the fitting parameters depends on the bacterial strain and the other only on the surface properties of the substrate.

## 1. Introduction

Orthopedic replacements help increase life expectancy and improve health conditions, especially in elderly patients. The progress in implant design is constant. Better mechanical properties and biocompatible surfaces make the devices more reliable. However, implant-associated infections are an unavoidable risk. Open fractures, contamination of the operating room or the patient's own skin or distal infections favor the presence of bacteria in the implant area [1]. In such a case, the likelihood of infection is greater if abiotic material is present than if no foreign material is involved. In fact, Elek and Conen [2] demonstrated in volunteers that local infections can be achieved with a bacterial concentration 10,000 times lower in the presence than in the absence of a synthetic material.

Colonization of implants by bacteria is a complex issue not fully understood. It is described that planktonic bacteria that get close enough to a material, due to their own motility or Brownian motion, can feel the surface and begin to land on the material. First long-range and then short-range forces, as van der Waals, electrical, hydrophobic, steric, and acid-base interactions drive the initial fixation of bacteria to the surface. Once on the surface, bond aging and biochemical interactions begin to play an important role through the polymeric structures present in the bacterial surface, strengthening the position of bacteria on the surface [3]. It is noteworthy that the adhesion of bacteria to surfaces triggers metabolic changes that make different the behavior of planktonic and sessile bacteria [4–6]. For some bacteria, the ability to produce exopolymeric substances (EPS) when they are on a surface is important. EPS sticks bacteria together and to the surface, helping the formation of biofilms. Biofilm provides an environment where bacteria are protected against external aggressions of the immune system or antibiotics. Its structure allows for some flow of nutrients and antibiotics, but as bacteria within the biofilm can be maintained with different metabolic activities, the effectiveness of antibiotics against bacteria in the biofilm decreases [7, 8]. Virulence factors of bacteria are diverse. In the case of the most frequent microorganisms involved in early orthopedic infections, *Staphylococcus aureus* and *Staphylococcus epidermidis*, their virulence factors are different. While *S. aureus* produces a variety of toxins and adherence factors, the main virulence factor for *S. epidermidis* comes from its ability to produce EPS to adhere to biomaterials and form biofilms [9–13].

Avoiding the infectious process in the presence of a biofilm is a difficult challenge due to the stable and strong fixation to the surfaces that the biofilm provides to the bacteria. Since the elimination of biofilms is problematic, a plausible method to avoid infections is to circumvent the formation of biofilms by acting on the first interactions between planktonic bacteria and the surface. If the surface is modified to reduce the initial adhesion of bacteria, the appearance of biofilms can be expected to decrease [14]. Therefore, it is of primary interest to obtain information on the behavior of the adhesion process during the first period of exposure of the surface to bacteria.

The density of bacteria adhered during the initial phase is expected to be related to the physical-chemical interactions between the surface and the bacteria, like what happens with abiotic colloidal particles [15]. Several models have been developed to predict the behavior of this initial process, from the most schematic, assimilating bacteria with hard and bare particles to a more elaborate analysis considering them as soft particles with protuberances [16]. Other studies relate the bacteria of a culture with a mixed system of different colloids, as they have shown that subpopulations in the same bacterial culture may have different adhesion behavior due to the different degree of superficial heterogeneity between the bacteria of that same culture [17]. However, a holistic perspective of the process is given by monitoring the temporal variation in bacterial coverage on the surface [3]. Parameters related to the kinetics of adhesion can provide relevant information on the colonization process. Among them, the bacteria-substrate affinity, defined as the rate at which bacteria adhere to the surface during the first instants of contact between the material and the bacterial suspension [18]. Several authors have analyzed the initial affinity between bacteria and substrates and, in many cases, this information provided a good clue for predicting the ability of a given material to resist bacterial colonization [18–23].

There are various experimental approaches to obtaining this information. However, an overall assessment of these interactions is best obtained when suspended bacteria flow in a laminar regime over the surface [24]. There are many studies on the initial adhesion of bacteria to surfaces using different flow chamber designs. There are flow chambers that allow direct observation of the adhesion process on transparent or translucent or highly reflective materials [18, 25–33]. For other materials, samples must be removed from the chamber to stain bacteria for observation [34, 35].

Titanium and its alloys are used for orthopedic implants as they meet many of the requirements to allow their use in bone replacement. Ti6Al4V improves the resistance of commercially pure titanium increasing the elastic modulus, the maximum tensile strength, the fatigue strength, and the corrosion resistance [36]. In a previous publication, we presented a new process for the functionalization by cross-linked aminosilanization of Ti6Al4V that improves its biocompatibility [37]. In that study, we found that the coating developed protects Ti6Al4V against bacterial adhesion under static conditions. However, more relevant information on the initial interaction between the surface and the bacteria can be obtained if the adhesion takes place under flow. For this purpose we will follow the adhesion process on this material for two strains of *S. epidermidis* which that are expected to produce different biofilms due to their different EPS production capacity.

## 2. Materials and Methods

Unless otherwise stated, all reagents were purchased from Panreac Quimica S.A.U., Barcelona, Spain. Deionized water used in this study was obtained by purification with a Milli-Q Direct water system (Millipore, Madrid, Spain).

### 2.1. Substrates

Ti6Al4V disks (ELI grade 23) 25 mm in diameter and 2 mm in thickness were obtained from DKSH Ltd (Switzerland). According to the manufacturer, this is the chemical composition (wt. %) of the bars from which disks were obtained: Al (6.2–6.1%), V (3.99–4.06%), Fe (0.083–0.082%), C (0.01–0.02%), N (0.016–0.014%), H (0.003%), O (0.07–0.06%) and Ti (balance). Manufacturer also specifies these mechanical properties: tensile strength: 890/920 MPa, yield strength 0.2%: 810/845 MPa, elongation: 19/20% and reduction of area: 49/52%. Samples of Ti6Al4V were abraded with silicon papers (Buehler P320, 300 rpm, 2 min), mechanically polished with diamond paste (9 *µ*m, 150 rpm, MetaDi Fluid, 10 min), and with colloidal silica (150 rpm, MasterMet2, 10 min). Then, disks were repeatedly rinsed and sonicated in deionized water (Milli-Q system), acetone and finally ethanol for periods of 10 min each. At the end, samples were dried at 40°C for 30 min and stored under vacuum for 12 h.

Disks of Ti6Al4V treated (T) were prepared from non treated (NT) disks subjected to an aminosilanization procedure, according to a methodology previously described [37]. In short, polished disks were subjected to chemical oxidation by treatment in a piranha solution (98% H_2_SO_4_ and 30% aqueous H_2_O_2_, 10 mL disk^−1^) at 45°C for 30 min. Samples, denoted as NT were not subjected to any further treatment. A second set of samples, referred as “treated” were then immersed into a closed crystallizer containing a 1 M solution (10 mL disk^−1^) of 3-(aminopropyl)trimethoxysilane (APTMS) in wet toluene ([H_2_O] ∼8 × 10^−2^ M). The reaction mixture was kept with stirring at 100 rpm, heated at 65°C until optimal coverage as inferred from the ninhydrin assay (usually 6 h). The disks were rinsed under flowing anhydrous toluene, then sonicated twice in the same solvent (30 mL disk^−1^) for 10 min each, and dried under air. Disks were thermally cross-linked at 120°C during 24 h, then immersed in phosphate buffered solution (PBS, 20 mL disk^−1^) at room temperature under stirring (700 rpm) for 48 h. Finally, disks were rinsed with distilled water and dried under a stream of Ar.

All samples were treated together in order to minimize the heterogeneity. Consequently, experimental uncertainty in respect to hydrophobicity is low. Measured water contact angle on NT and T surfaces were 48 ± 2° and 83 ± 2°, respectively [37].

### 2.2. Bacterial Strains and Culture

The strains used in the testwere* S. epidermidis* ATCC35983 (*S. epidermidis3*), medium EPS producer, and *S. epidermidis* ATCC35984 (*S. epidermidis4*) high EPS producer. These strains differ in their capacity of segregating polysaccharide intercellular adhesin when they are in a growth media [38, 39]. The strains were stored at −80°C in porous beds (Microbank, Pro-Lab Diagnostics, USA). Cultures were obtained from blood agar plates where bacteria from the frozen stock were inoculated and incubated at 37°C. From these cultures, tubes of 4 mL of Trypticase Soy Broth (TSB) (BBL, Becton Dickinson, USA) were incubated for 10 h and maintained at 37°C. Then 25* µ*L of this preculture was used again to inoculate 50 mL flasks of TSB at 37°C for 14 h. This time was enough to guarantee that strains were at the end of the exponential phase of growth, as it was checked with their growth curves that had been previously carried out. The bacteria were then harvested by centrifugation for 5 min at 1000 rpm (Sorvall TC6, Dulont, USA) and washed three times with 0.15 M phosphate buffered saline, PBS, (0.87 g·L^−1^ of K_2_HPO_4_, 0.68 g·L^−1^of KH_2_PO_4 _and 8.76 g·L^−1^of NaCl) pre-conditioned at 37°C. Afterwards, bacteria were re-suspended in a volume of PBS enough for the whole adhesion experiment, at a concentration of 3 × 10^8^ bacteria mL^−1^. Along the test, this stock suspension was actively stirred to ensure a constant bacterial concentration.

### 2.3. Bacterial Adhesion

**Figure 1:**
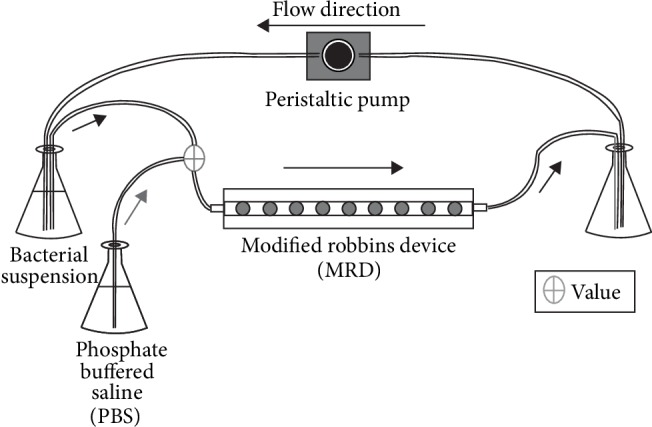
Scheme of the experimental setup for the adhesion experiments using a modified Robbins device (MRD).

Adhesion experiments were carried under flow using a Modified Robbin Device (MRD).  Figure 1 shows a scheme/diagram of the MRD with the experimental setup. This flow chamber allows testing several samples under the same bacterial suspension. The MRD is a pipe of rectangular section, along which the samples are placed. The flow inside the MRD was kept at a constant rate of 2 mL·min^−1^. Under these conditions, flow was in laminar regime, the Reynolds number was 1.64, the wall shear rate was 0.97 s^−1^, and the shear stress was 6.8 10^−4^ N·m^−1^. Samples were exposed to the bacterial flow using supports screwed through one of the walls of the MRD, ensuring they were perfectly leveled to avoid any turbulence of the flowing liquid. The MRD used allowed testing nine different samples simultaneously. Any support with a sample can be extracted from the device at any time and replaced with other support with a dummy surface attached.

Once all the samples were in their position in the MRD, PBS was let to flow for 15 min. Then, the flow was gently switched to the bacterial suspension, and after 5, 10, 15, 20, 30, 40, 60, and 90 min, one sample at each time was removed. Extracted samples were passed by a PBS solution and then dried in a sterile environment. Afterwards, bacteria on the surface were stained with SYTO9 (Invitrogen SA, Spain), observed by epifluorescence microscopy and automatically counted with the software NIS-Elements BR 4.10 (Nikon Instruments INC., USA). Counting on each sample was done on at least 9 places randomly selected. All the experiments were done at 37°C, and repeated at least three times with independent cultures. For each of the times tested, the samples were removed from different positions in the MRD in each of repetition of the experiment.

Due to the complexity of the experimental procedure, reproducibility tests of the whole experiment were carried out. One aspect that needs consideration is the distance of each sample to the flow entrance, since the samples are in line in the same direction than flow. Some studies have found dependence on this distance, but positive or negative depending on the liquid velocity [40]. Another aspect that needs attention deals with the procedure of extraction and measurement of the number of bacteria on each surface. When samples are removed from MRD, their surface passes from liquid to air. That passage can remove from the surface bacteria not enough attached to surmount the force acting on them by surface tension. In the control experiments, all the samples were under the same bacterial flow during the same time. Then, samples in even positions in the MRD were removed and replaced by dummy samples. Next, an air bubble was injected through the inner channel of the MRD that implies the passage of two air-liquid interfaces over the surfaces that could sweep bacteria from the samples, simulating the force suffered in extraction. Afterwards, samples in the odd positions were removed. Finally, quantification of adhered bacteria on each surface was done as described previously. These experiments were triplicated, and the number of adhered bacteria per unit area were comprised between (43 ± 2) × 10^4^ bacteria·cm^−2^ and (45 ± 3) × 10^4^ bacteria·cm^−2^in odd positions and (42 ± 3) × 10^4^ bacteria·cm^−2^ and (46 ± 3) × 10^4^ bacteria·cm^−2^ in even positions. This test ensures that our results are reproducible within an uncertainty level lower than 10%.

### 2.4. Statistical analyses

The data are expressed as means ± standard deviation. Differences between the means were determined by a one-way analysis of variance (ANOVA) after confirmation of data normality using IBM SPSS Statistics v19 (IBM company, New York, USA). Statistical significance was accepted for *p* < 0.05 after comparing the mean values by the Tukey HSD test.

## 3. Results and Discussion

**Figure 2:**
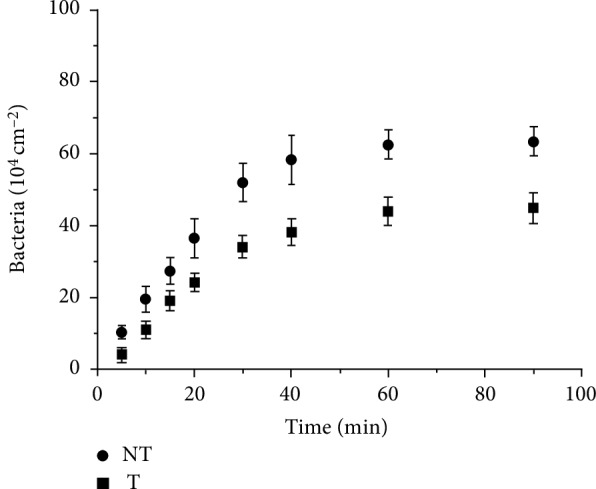
Temporal evolution of *S. epidermidis*3 adhesion on treated (T) and nontreated (NT) surfaces.

**Figure 3:**
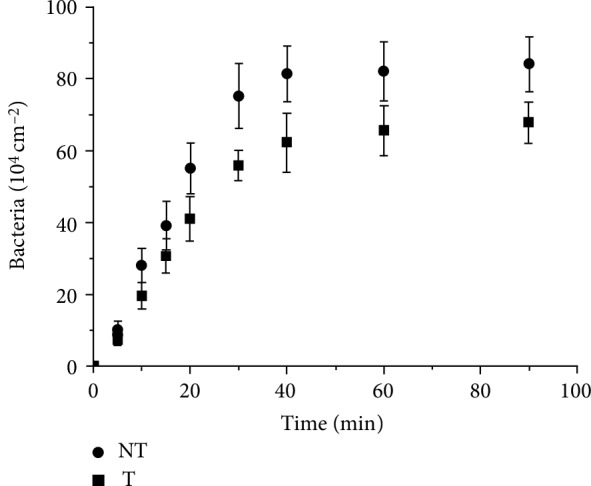
Temporal evolution of *S. epidermidis*4 adhesion on treated (T) and nontreated (NT) surfaces.

**Figure 4:**
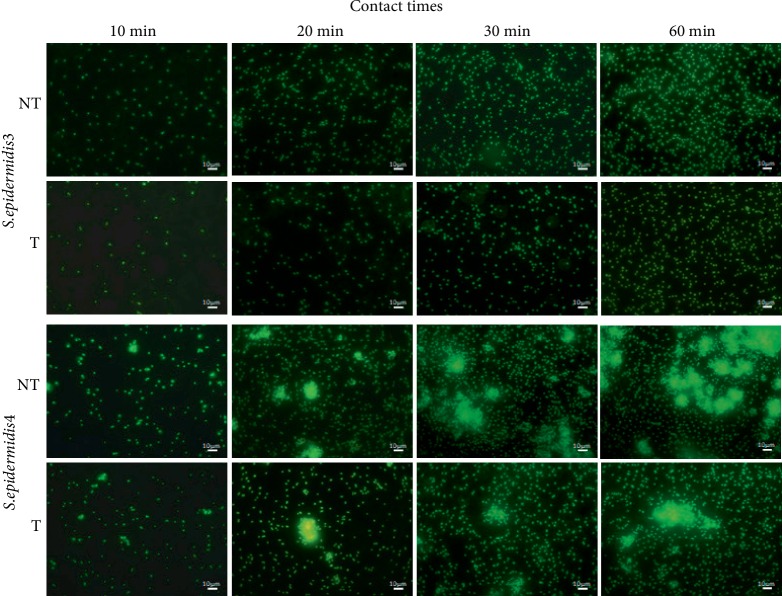
Fluorescence images****of typical areas on treated (T) and nontreated (NT) surfaces as function of the contact time under flow conditions for *S. epidermidis*3 and *S. epidermidis*4.

Once a material is put in contact with a bacterial suspension, microorganisms begin the colonization of the surface. This process happens for stagnant as well as flowing bacterial suspensions. Figures 2 and 3 show the time dependence of the surface density of retained bacteria on the treated (T) and the nontreated (NT) surfaces, for *S. epidermidis*3 and *S. epidermidis*4, respectively. In all cases, the adhesion rate is depending on the time elapsed since the beginning of the flow, decreasing as the coverage of bacteria increases. This behavior can be related to a gradual occupation of surface positions by bacteria, masking an area that could account up to thirty times its geometrical section, as for abiotic particles [41], making difficult for newcomers the access to the surface. Both strains adhere more on the NT than on the T surfaces, but, for each surface condition, the retention of *S. epidermidis*4 is higher than for *S. epidermidis*3.  Figure 4 presents various images of typical areas on treated (T) and nontreated (NT) surfaces as function of the contact time to better visualize the attachment of bacteria under flow conditions.

Modelling bacteria as colloidal abiotic particles provides successful approaches to analyze bacteria-surface interplay. Visualization of samples after 90 min of adhesion (images not shown) exhibit bacteria well dispersed on the surface. Assuming microorganisms as colloidal particles, bacterial retention could fit a model in which the number of adhered bacteria depends on the available positions on the surface, with no contribution of bacterial co-adhesion. This model, schematically *A* + *B* → *AB*, is associated to a pseudofirst order kinetic that assumes the rate of surface coverage as proportional to the free positions on the surface:

(1) $$\begin{array}{c} \frac{d\theta \left(t\right)}{dt}=k\left(1-\theta \left(t\right)\right). \end{array}$$

being θ(t) the surface coverage, defined as *θ*(*t*) = *n*(*t*)/*n*_*∞*_; *n*(*t*) the bacterial density on the surface at time t, and *n*_*∞*_ its limit value at very long time. *k* is a proportionality constant that should be related to the retention energy of bacteria on the surface. Integration of Equation (1) leads to

(2) $$\begin{array}{c} n\left(t\right)=n_{\infty }\left(1-e^{-kt}\right). \end{array}$$

Table 1 includes the values of *n*_*∞*_ and *k* obtained for each of the conditions studied. For each surface, *n*_*∞*_is lower for *S. epidermidis*3 than for *S. epidermidis*4. Also, for each bacterial strain, *n*_*∞*_ is lower on the treated surface than on the nontreated one, being this reduction equals to 29% for *S. epidermidis*3 and 19% for *S. epidermidis*4.

From Equation (2) it is also possible to evaluate the interaction between bacteria and substrate at the very initial stages of the temporal evolution of the adhesion, or bacterium-substrate affinity, *j*_*o*_, according to:

(3) $$\begin{array}{c} j_{o}=\underset{t\to 0}{\lim}j\left(t\right)=\underset{t\to 0}{\lim}\frac{dn\left(t\right)}{dt}. \end{array}$$

Table 1 shows the values of *j*_*o*_ obtained. Affinity results points at the same behavior than shown by *n*_*∞*_. For any of the surface finishes, affinity is higher for *S. epidermidis*4 than for *S. epidermidis*3. Also, for each of the bacterial strains, affinity is lower for T than for NT surfaces, ranging from (1.7 ± 0.3) × 10^4^ cm^−2^·min^−1^ to (4.1 ± 0.6) × 10^4^ cm^−2^·min^−1^. This similar behavior is also observed in other systems. Progress of the adhesion of S*. epidermidis4* and other bacterial strains on surfaces of stainless steel and Ti6Al4V that were submitted to different treatments, show also a direct correlation between the affinity, *j*_*o*_, and the limit value of the number of adhered bacteria, *n*_*∞*_ [21–23].

Assumption of bacteria as colloidal particles is accepted as a model to obtain information of the real behavior of these microorganisms on surfaces. Sjollema et al. [42] evaluated the deposition rate of a flowing suspension of colloidal particles, *j*_*SL*_, according to the Smoluchowski-Levich approach given by

(4) $$\begin{array}{c} j_{SL}=\frac{DC}{a}Sh, \end{array}$$

where *C* is the concentration of particles of radio a in the suspension, *D* is the Stoke-Einstein diffusion coefficient and *Sh* the Sherwood number [43]

(5) $$\begin{array}{c} Sh=\frac{1}{\Gamma \left(4/3\right)}{\left(\frac{2bP_{e}}{9x}\right)}^{1/3}, \end{array}$$

being *x* the distance from the flow inlet and b the half-depth of the MRD, and *P*_*e*_ the Pèclet number given by *P*_*e*_ = 3*va* ^3^/2*b* ^2^ *D*, being v the fluid velocity. For our experimental conditions, *P*_*e*_ equals to 6.2 × 10^−5^,and *Sh* equals to 7.9 × 10^−3^.

Then, from Equation 4, the theoretical upper limit for the initial deposition rate, *j*_*SL*_, obtained is 1.24 × 10^4^ bacteria·cm^−2^·min^−1^, lower than any of the *j*_*o*_ experimental values obtained (Table 1). Other authors also found discrepancies between the theoretical and the experimental deposition rates for bacterial suspensions, who related to the failure on the fulfillment of conditions for the application of the Smoluchowski-Levich approach, due to the radius and/or to possible appendages of bacteria protruding from the surface [18, 44].

Nevertheless, it can be expected that differences in surface properties between strains imply also different behavior against surfaces, so it should be expected that different behavior not only depends on the strain but also on the alloy surface. The properties of the surface modify the deposition rate also for abiotic particles, as found by Dabroś and van de Ven for polystyrene particles on cover glass slides, where the deposition rate was found to be dependent on the cleaning method of the slides and conditioned to interactions at separations smaller than 10 nm [41].

To find as much clear as possible the dependence on both factors of adhesion, namely surface characteristics of the strain and surface characteristic of the material, it is of interest following the evolution with time of the actual experimental surface coverage, *θ*_*e*_(*t*) = *n*(*t*)/*n*_90_. In this way, the fitting parameters in a pseudofirst order kinetic equation can be used to modulate the exponential behavior of the coverage. Then, taking the maximum experimental bacterial density *n*_90_ as a reference for each experiment, the bacterial retention could be described as modified pseudofirst order kinetics, according to

(6) $$\begin{array}{c} 1-\theta_{e}\left(t\right)=ge^{-k_{e}t}. \end{array}$$

This equation is similar to Equation (2), but being *g* and *k*_*e*_ adjustable parameters instead of *n*_*∞*_ and *k*. The values obtained for *g* and *k*_*e*_ are listed in Table 2. In all cases, *k*_*e*_ is larger than *k* and *g* is larger than one. It is remarkable that it appears that *g* and *k*_*e*_ are uncoupled, each of them from the surface characteristics and the microorganisms, respectively. The obtained values of *g* depend only on the bacterial strain, in agreement with the proposal that discrepancies from the colloidal model are related to the bacterial characteristics [44]. Values of *g* are higher for *S. epidermidis*3 than for *S. epidermidis*4, that suggest worse initial effectiveness in adhesion of *S. epidermidis*3 than *S. epidermidis*4, irrespective of the surface finish. Surface charge is an important factor in the adhesive behavior of microorganisms. We have previously measured the zeta potential of both strains in PBS. The values were −(16 ± 8) mV for *S. epidermidis*3 (unpublished result) and −(6 ± 2) mV for *S. epidermidis*4 [21]. Nevertheless, despite a slightly higher zeta potential for *S. epidermidis3*, experimental uncertainties do not ensure that the electrical properties make a difference in the behavior of the two strains. However, EPS production appears to be a discriminating characteristic between both strains. *S. epidermidis*3 is a moderately EPS-producing strain in contrast with *S. epidermidis*4 that produces EPS in a large extent. This is reflected in their surface characteristics. An AFM study showed that *S. epidermidis*4 is fully covered by a slime layer with higher adhesion to the tip than the slime for *S. epidermidis*3 which is only partially slime-covered [39]. Song et al. [45] studied the vibration of some bacterial cells adhered on glass under static and flow conditions. They found that the vibrational amplitude of *S. epidermidis*3 is significantly higher than for *S. epidermidis*4, being the higher vibrational amplitudes associated to smaller spring constants. This behavior suggests a firmer and better localized adhesion of *S. epidermidis*4, being more closely related to the irreversible adhesion process given by *A* + *B* → *AB* given by a g value equal to 1.

On the other hand, the exponential parameter *k*_*e*_ relates the surface availability and the rate of the coverage. This means that for a given coverage, as much larger the *k*_*e*_ parameter is, the larger the rate of surface occupation will be. For a given occupation of the surface, it reflects the capability of the surface of the material to attract new particles that in turn is related to the properties of the material. In the present study, the treatment applied to the surface of Ti6Al4V yields a *k*_*e*_ lower than for the original surface. That suggests that the coverage provided to samples protects in some extension the alloy material from bacterial colonization. That protection was observed in static experiments [37]. The amino groups introduced in surface, carrying positive charge, could favor adhesion of negatively charged bacteria. However, this attractive contribution is not enough to surpass its capability to conceal the affinity of bacteria for the surface of Ti6Al4V, enriched in OH groups by the piranha solutions, previous to the silanization process. Interestingly, this protection against bacteria resists flow conditions.

Using Equation (3), the initial slope, *j*_*oe*_, of the process described by Equation (6) can be evaluated as *j*_*oe*_ = *n*_90_.*g*.*k*_*e*_. This new parameter is listed in Table 2. In all cases, *j*_*oe*_ is higher than *j*_*o*_. The higher value of *j*_*oe*_ is consequence of the short difference between *n*_*∞*_ and *n*_90_, despite the larger differences in the time associated to each of these two bacterial densities. Interestingly, it appears that, within the experimental uncertainty, *j*_*oe*_ is independent of the bacterial strain, no matter the large differences of the coverages after 90 min, *n*_90_, of both strains. This result suggests that the *j*_*oe*_ parameter provides a clearer gauge than *k*_*e*_ for the adhesive behavior of the surfaces against the *S. epidermidis* strains tested.

## 4. Conclusions

The use of a modified Robbins device allows the monitoring of bacterial adhesion on Ti6Al4V with different surface treatment. Bacteria tested differ on their ability to produce EPS. From the dynamic adhesion information provided using the Robbins device, an analysis based on a pseudofirst order kinetics allows to discriminate the behavior of each surface and each strain on the surfaces. Since the calculated parameter *g* can be related to the strain, the *j*_*oe*_ parameter reports only from the adhesion characteristics of the Ti6Al4V surface. Additional studies will allow us to extend this analysis to other groups of bacteria and/or surface modifications.

## Figures and Tables

**Table 1:** Fitted parameters from Equations 2 and 3, *n*_*∞*_, *k*, and *j*_*o*_, to the treated (T) and nontreated (NT) surfaces for *S. epidermidis*3 and *S. epidermidis*4.

Surface-strain	*n*_*∞*_	*k*	*j*_*o*_
	(bact cm^−2^ × 10^4^)	(min^−1^)	(bact cm^−2^ min^−1^ × 10^4^)
NT-*S. epidermidis*3	69 ± 3	0.039 ± 0.004	2.7 ± 0.4
T-*S. epidermidis*3	49 ± 3	0.034 ± 0.003	1.7 ± 0.3
NT-*S. epidermidis*4	91 ± 6	0.045 ± 0.004	4.1 ± 0.6
T-*S. epidermidis*4	74 ± 4	0.040 ± 0.005	3.0 ± 0.5

**Table 2:** Fitted parameters from Equations (6) *gk*_*e*_, and *j*_*oe*_, to the treated (T) and nontreated (NT) surfaces for *S. epidermidis*3 and *S. epidermidis*4.

Sample-strain	*n*_90_	*g*	*k*_*e*_	*j*_*oe*_
	(bact cm^−2^ × 10^4^)		(min^−1^)	(bact cm^−2^ min^−1^ × 10^4^)
NT-*S. epidermidis*3	63 ± 3	1.58 ± 0.15	0.075 ± 0.014	7 ± 2
T-*S. epidermidis*3	45 ± 2	1.57 ± 0.03	0.067 ± 0.003	4.7 ± 0.5
NT-*S. epidermidis*4	85 ± 6	1.29 ± 0.12	0.074 ± 0.008	8 ± 2
T-*S. epidermidis*4	68 ± 3	1.30 ± 0.10	0.064 ± 0.005	5.7 ± 1.1

## Data Availability

The data used to support the findings of this study are included within the article.
